# Global Health Diplomacy: A Closer Look

**Published:** 2019-08

**Authors:** Rahim KHODAYARI-ZARNAQ, Gisoo ALIZADEH, Neda KABIRI

**Affiliations:** Iranian Excellence Center for Health Management, School of Management and Medical Informatics, Tabriz University of Medical Sciences, Tabriz, Iran

## Dear Editor-in-Chief

Outbreak of some diseases such as HIV/AIDS, Severe Acute Respiratory Syndrome (SARS), and Ebola Virus has created a demand for making appropriate policies and diplomatic coordination in the international level and has turned international health to the core component of foreign policy in the recent decades (1). Peter Bourne, Jimmy Carter’s special advisor for health affairs, introduced “Medical Diplomacy” for the first time in 1978. Improvements in international communications between policy makers and researchers changed this concept to “Global Health Diplomacy”, the concept which contains performances of public and private actors in order to improve global health (2). “Global Health Diplomacy” was applied operationally in 1851, when European Unions had come together for coordination of some diseases like Cholera, Plague, and Yellow Fever (3). There are different definitions of “Global health diplomacy” in the literature. One of the most comprehensive definitions relates to Adams and Novotny (4) in which global health diplomacy is a political change in order to achieve intrinsic goals of health promotion through strengthening international relationships especially in areas with resource constraints. Health diplomacy was noted as a means to protect you in the global society as well as an opportunity for bridging gap among governments, private sector, and non-governmental organizations in order to improve public health (5).

In recent decades, some health policies have succeeded in increasing political reputation or improving relations between states and political actors. Among these programs as soft power, some countries as China and Cuba send a large number of physicians to the African countries. Establishment of hospitals and health care centers in these countries is another type of soft power policies (6). Given the importance of health in sustainable development, health diplomacy needs to be one of the main tools of foreign policy in countries. Moreover, health diplomacy should be considered more increasingly by governments to achieve their superior health goals in their societies.
